# Novel technologies combined with traditional metabolic engineering strategies facilitate the construction of shikimate-producing *Escherichia coli*

**DOI:** 10.1186/s12934-017-0773-y

**Published:** 2017-09-29

**Authors:** Pengfei Gu, Xiangyu Fan, Quanfeng Liang, Qingsheng Qi, Qiang Li

**Affiliations:** 1 School of Biological Science and Technology, University of Jinan, Jinan, 250022 People’s Republic of China; 2 State Key Laboratory of Microbial Technology, Shandong University, Jinan, 250100 People’s Republic of China

**Keywords:** Shikimate, *Escherichia coli*, Metabolic engineering, Novel technologies

## Abstract

Shikimate is an important intermediate in the aromatic amino acid pathway, which can be used as a promising building block for the synthesis of biological compounds, such as neuraminidase inhibitor Oseltamivir (Tamiflu^®^). Compared with traditional methods, microbial production of shikimate has the advantages of environmental friendliness, low cost, feed stock renewability, and product selectivity and diversity, thus receiving more and more attentions. The development of metabolic engineering allows for high-efficiency production of shikimate of *Escherichia coli* by improving the intracellular level of precursors, blocking downstream pathway, releasing negative regulation factors, and overexpressing rate-limiting enzymes. In addition, novel technologies derived from systems and synthetic biology have opened a new avenue towards construction of shikimate-producing strains. This review summarized successful and applicable strategies derived from traditional metabolic engineering and novel technologies for increasing accumulation of shikimate in *E. coli*.

## Background

Shikimate is a hydroaromatic intermediate compound in microorganisms, which is the precursor of three aromatic amino acids, l-tryptophan, l-phenylalanine, and l-tyrosine [1]. Nowadays, more and more attention has been attracted on the synthesis of shikimate as it is a promising building block for other important aromatic compounds with pharmaceutical activities, such as salicylic acid, alkaloid, flavonoid, coumarins, and violacein [2]. It is also the key synthetic material of neuraminidase inhibitor Oseltamivir (marketed as Tamiflu), which can inhibit the release of new virus particles of influenza B, H1N1 and H3N2 from the infected cells [3]. As the outbreaks of human and avian influenza viruses, the market price of shikimate increased from the usual price of $40/kg to $1000/kg due to a huge demand for Tamiflu [3].

The production of shikimate is mainly dependent on a conventional, low-yielding extraction from the seed of *Illicium* plant, such as *I. verum* and *I. anistatum*. However, this method is low-yield and costly, and cannot meet the increasing demand of shikimate [4]. In contrast, microbial production of shikimate from renewable resources like glucose is an alternative and sustainable approach to meet the current market volume.

**Fig. 1:**
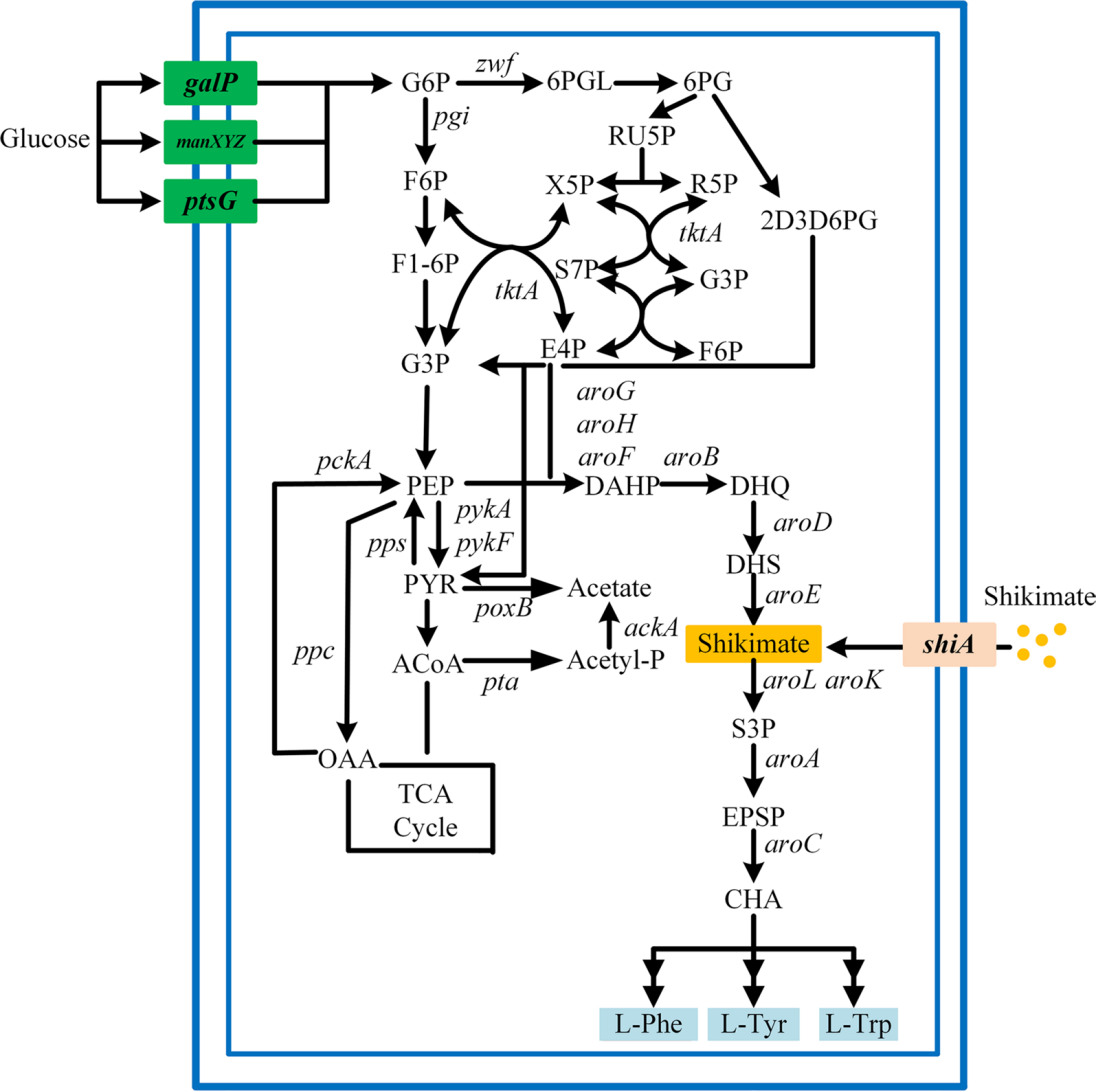
The schematic of shikimate pathway in *E. coli*

Because of clear genetic background, fast growth in inexpensive media, and easy genomic manipulation, *E. coli* is a preferred candidate host for shikimate production [5, 6]. In *E. coli*, the shikimate pathway starts from the condensation of phosphoenolpyruvate (PEP) from glycolysis and d-erythrose 4-phosphate (E4P) from pentose phosphate pathway, which is catalyzed by three different DAHP synthase isoenzymes AroF, AroG and AroH. In addition, these three isoenzymes are feedback inhibited by three aromatic amino acids, l-tyrosine, l-phenylalanine, and l-tryptophan, respectively. And then, DAHP is converted into 3-dehydroquinate (DHQ), 3-dehydroshikimate (DHS), and shikimate in turn, catalyzed by dehydroquinate synthase encoded by *aroB*, DHQ dehydratase encoded by *aroD*, and shikimate dehydrogenase encoded by *aroE* respectively. Subsequently, by shikimate kinase isoenzymes AroK and AroL, and EPSP synthase AroA, a common precursor chorismate can be generated from shikimate, which proceeds into the biosynthetic pathway of three aromatic amino acids. The schematic of shikimate pathway in *E. coli* was illustrated in Fig. 1.

Through decades of efforts, the shikimate pathway has been well elucidated and characterized [7, 8]. With the help of tools derived from metabolic engineering, system biology and synthetic biology, a particular pathway can be rationally engineered to achieve high titer, yield and productivity of targeted chemicals [9–11]. In previous work, our group also utilized metabolic engineering and synthetic biology strategies to construct high producing strains of l-tryptophan, shikimate, succinate, and 5-aminolevulinic acid [12–16]. In this review, we summarized a plenty of traditional metabolic engineering strategies and novel technologies that improving the production of shikimate in *E. coli*.

## Traditional metabolic engineering strategies for improving the titer of shikimate in *E. coli*

### Increasing the intracellular level of precursors

In *E. coli*, PEP acts as a phosphoryl group donor in cross-membrane transportation of glucose by phosphoenolpyruvate: carbohydrate phosphotransferase system (PTS). When 1 mol of glucose is transported into the cell, 1 mol of PEP is consumed, resulting in PTS the largest consumer of PEP. It was reported the relative flux of PEP directed into shikimate pathway is only around 1.5% of the PTS system consumed [17]. Therefore, inactivating the PTS system will increase the proportion of PEP which entered the shikimate pathway [6]. However, low consumption rate of glucose may exhibit when PTS is abolished. To solve this problem, PTS can be replaced with other glucose transport systems in which PEP is not consumed, such as native galactose permease (GalP) [18, 19], and glucose facilitator (Glf)/glucokinase (Glk) from *Zymomonas mobilis* [20]. Another approach to increase PEP supply is overexpression of PEP synthase (PpsA) to recycling pyruvate to PEP [5]. In addition, to bypass the limited PEP availability, Ran et al. utilized pyruvate, rather than PEP, to make the shikimate pathway intermediate DAHP [21]. E4P, another precursor of shikimate, is derived from the pentose phosphate pathway. To improve the intracellular concentration of E4P, over-expression of transketolase and transaldolase encoded by *tktA* and *talB* respectively is a common strategy [20, 22]. As NADPH was also involved in shikimate synthesis, overexpression of transhydrogenase encoded by *pntAB*, and NAD kinase encoded by *nadK* will increase the size of NADPH pool, and in turn increase shikimate production [23].

### Blocking downstream pathway of shikimate

In wild type *E. coli*, to synthesize downstream aromatic amino acids and other aromatic compounds indispensable for cell, shikimate cannot be accumulated. Accordingly, inactivation of downstream pathway after the stage of shikimate is employed for engineering shikimate-producing strain. The shikimate kinase I and II, encoded *aroK* and *aroL* respectively, were often selected to perform gene disruption [6, 24]. As shikimate-3-phosphate can easily transformed into shikimate by heating or acidification, accumulation of shikimate may also be accomplished by inactivating *aroA* in *E. coli*. This strategy has been explored in *B. subtilis*, and accumulation of 1.1 g/L shikimate was exhibited in the final strain [25].

### Overexpression of rate-limiting enzymes in the shikimate pathway

In shikimate pathway of *E. coli,* three rate-limiting enzymes are involved, comprising DAHP synthase isoenzymes encoded by *aroF*, *aroG*, and *aroH*, 3-dehydroquinate synthase encoded by *aroB*, and shikimate dehydrogenase encoded by *aroE*, respectively [2, 24]. Among them, the activity of DAHP synthase is crucial which determines the carbon flux directed into shikimate pathway [26]. In wild-type *E. coli*, DAHP synthase isoenzymes AroF, AroG, and AroH can be feedback inhibited by three aromatic amino acids, l-tyrosine, l-phenylalanine, and l-tryptophan separately. It was reported AroF and AroG respectively contribute 20 and 80% of the total enzyme activity [27]. Therefore, these two isoenzymes were often employed as a target for site-directed mutagenesis and overexpression [6, 28]. In addition, AroB and AroE are both feedback regulated by shikimate. It was reported deletion of ShiA transporter was effective for decreasing the intracellular shikimate level and maintaining the regular activities of AroB and AroE [29, 30]. Considering the drawbacks of plasmids, site-specific integration of key genes into the chromosome of target strain could make genes stably expressed without inducers. In 2016, Liu et al. integrated crucial genes *aroG*, *aroB*, *tktA*, *aroE*, *glk*, *galP* into the locos of *ptsHIcrr*, and *ppsA* gene into the locos of *tyrR* [31]. With these modifications, the final strain produced 4.14 g/L shikimate in shake flask and 27.41 g/L in 5-L fermentator, of which titer was comparable with shikimate producers containing recombinant plasmids. Table 1 summarizes the shikimate production titer and yield of different recombinant *E. coli* strains reported in the last few years.

**Table 1:** Comparison of shikimate production in different recombinant *E. coli* strains

*E. coli* strains	Engineering strategies	Culture methods	Carbon source	Shikimate production		References
				Titer (g/L)	Yield (g/g)	
SP1.1*pts/*pSC6.090B	RB791 (*ΔptsHΔptsIΔcrr*, *serA*::*aroB*, *aroL478*::Tn*10*, *aroK17*::Cm^R^)/pSU18-*P*_*tac*_ *glfglk*-*aroF* ^*FBR*^-*tktA*-*P*_*tac*_ *aroE*-*serA*	10-L fed-batch fermentation	Glucose	87	0.348	[20]
SP1.1/pKD12.138	RB791 (*ΔptsHΔptsIΔcrr*, *serA*::*aroB*, *aroL478*::Tn*10*, *aroK17*::Cm^R^)/pSU18-*aroF* ^*FBR*^-*P*_*tac*_ *aroE*-*serA*-*tktA*	2-L fed-batch fermentation	Glucose	52	0.174	[22]
SA116	BW25113 (*ΔaroKΔaroLΔrecA*, *Ppps::PlacQ1, PcsrB::PlacQ1*,) containing *aroG* ^*FBR*^-*tktA*-*aroB*-*aroE* gene cluster integrated by CIChE and an additional chromosomal copy of *nadK*	Batch fermentation	Glucose	3.12	0.319	[23]
PB12.SA22	JM101 (*ΔptsHIcrrΔaroL ΔaroK,* glc^+^)/pJLB-*aroG* ^FBR^-*tktA* and pTOPO -*aroB*-*aroE*	1-L batch fermentation	Glucose	7.05	0.29	[6]
SA5/pTH-aroG^fbr^-ppsA-tktA	B0013 (*ΔaroLΔaroK, ΔptsGΔydiBΔackAΔpta*)/pTH18kr-*aroG* ^*FBR*^-*ppsA*-*tktA*	7-L fed-batch fermentation	Glucose	14.6	0.293	[5]
P-9	BW25113 (*ΔaraCΔptaΔptsGΔaroLΔtrpRΔpykF*) with the wild *aroK* promoter replaced by the tunable switch/pUC19-*aroE*-*aroD*-*aroB* and pCL1920-*aroG* ^*FBR*^-*tktA*	5-L fed-batch fermentation	Glucose	13.15	0.204	[12]
SA5/pGBAE	BW25113 (*ΔaroLΔaroK, ΔptsHIcrr*::*aroG*-*aroB*- *tktA*-*aroE*-*glk*-*galP*, *ΔtyrR*::*ppsA*)/pETDuet-1 -*P*_*T7*_-*aroG*-*aroB*-*P*_*T7*_-*tktA*-*aroE*	5-L fed-batch fermentation	Glucose and glycerol	27.41	N^a^	[31]
SK5/pSK6	BW25113 (*ΔaroLΔaroKΔydiBΔppcΔldhA*)/pSA40-*aroB*-*tktA*-*aroG* ^*FBR*^-DHQ/SDH-*rpsM*-*aroK*	Batch fermentation	Glycerol	5.33	N	[8]
DH5a-T7-P-DK	BL21 (DE3)/pET-28a-*glpD*-*glpK* and pAOC-*aroE*-*aroB*-*glk*-*tktA*-*aroF* ^*FBR*^	Batch fermentation	Glycerol	0.2	0.175	[44]
BW25113 (*ΔaroLΔaroK*, DE3)/pETDuet-GBAE	BW25113 (*ΔaroLΔaroK*, DE3)/pETDuet-1-*aroG*-*aroB*-*tktA*-*aroE*	Batch fermentation	Sorbitol	1.0776	0.192	[45]
DHPYAAS-T7	DH5α (*ΔptsHIcrr ΔaroLΔydiBΔaroK*)/pAOC-*aroE*-*aroB*-*glk*-*tktA*-*aroF* ^*FBR*^	10-L fed-batch fermentation	Glycerol	1.85	0.093	[24]

^a^Not indicated in the reference

## Novel technologies increased the production or stability of shikimate-producing strains

### Modified chemically inducible chromosomal evolution

As mentioned above, to achieve accumulation of shikimate, rate-limiting enzymes, such as *aroG*, *aroE* and *aroB* were often overexpressed by plasmids. However, due to structural instability, segregational instability, and allele segregation, genetic instability often exhibited for plasmids resulting in decreased productivity of the desired compound [32]. To maintain the stable existence of plasmids in host cells, antibiotic or other selective agents should be utilized, which increases the overall bioprocess cost and generates environmental concerns. Moreover, metabolic burden would be generated as the duplicate of plasmids competed for carbon source, energy and reduce power with the host. In other hand, although several chromosomal integration strategies have been extensively reported, little attention has been paid to the integration of gene(s) with multiple copies.

**Fig. 2:**
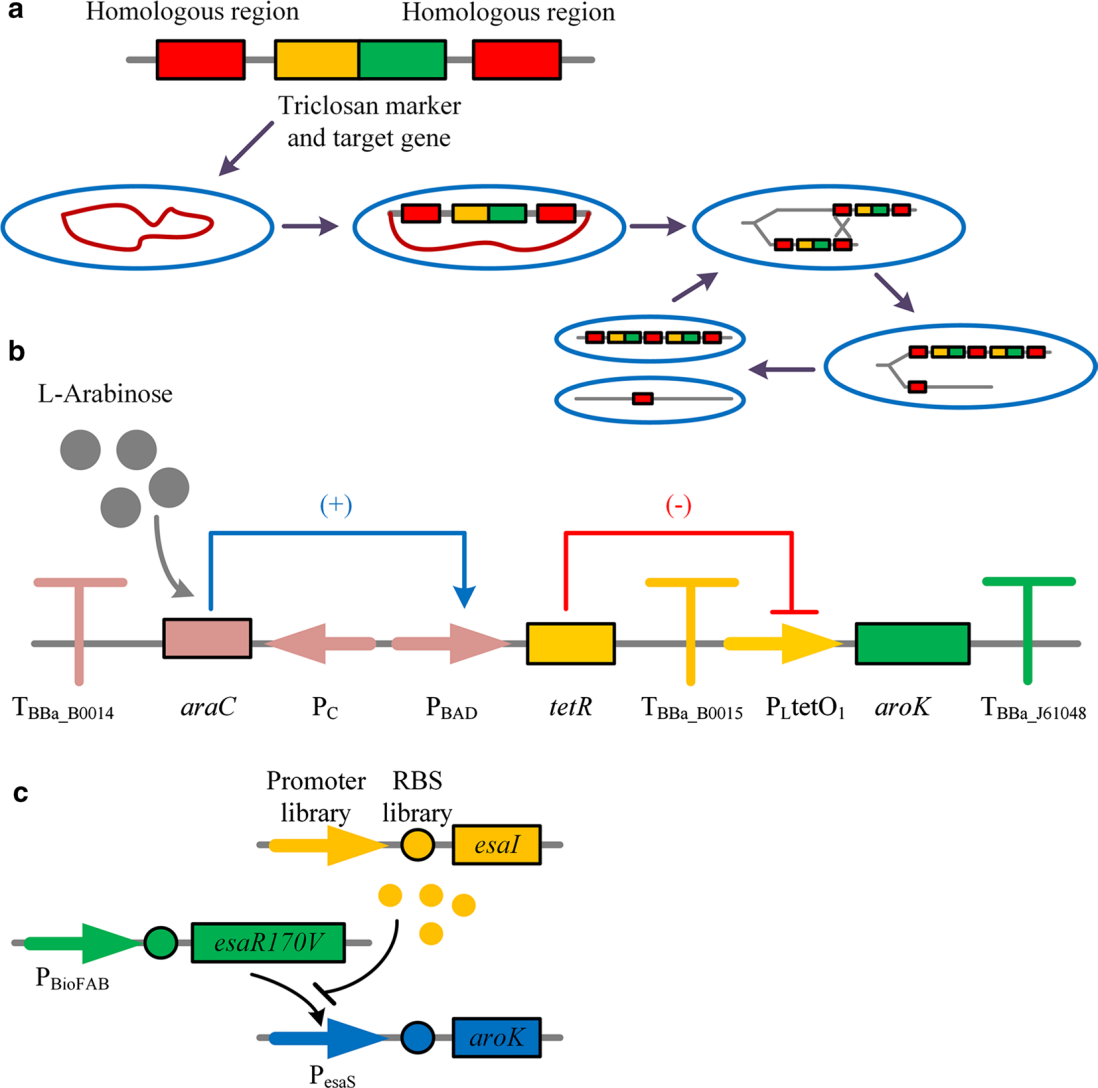
Novel technologies reviewed in this paper. **a** Modified chemically inducible chromosomal evolution (CIChE). The CIChE DNA cassette contains a triclosan marker and target gene(s), flanked by homologous regions. By *recA*-mediated recombination between the leading homologous region in one DNA strand with the trailing homologous region in another strand, one daughter cell contains two copies of the cassette will be generated. This process can be repeated when *recA* is present. **b** Tunable switch. When no inducer was added, the expression of TetR controlled by P_BAD_ promoter was repressed, leading to normal expression of *aroK* under the regulation of P_LtetO1_. When l-arabinose exists, P_BAD_ promoter was induced and transcription of P_LtetO1_ promoter was partially repressed by expressed TetR, resulting to decreased expression of *aroK*. **c** When AHL is absent, the transcriptional regulator EsaRI70V binds to the P_esaS_ promoter and transcription of *aroK* is activated. As the accumulation of AHL generated by AHL synthase EsaI, binding of EsaRI70V is disrupted eventually and the activity of P_esaS_ promoter is inhibited. As the expression level of EsaI can be varied by promoter and/or RBS libraries, the transcription of *aroK* can be regulated at variable times and cell densities during the fermentation

To overcome these drawbacks of plasmids and traditional genomic integration with single copy, Tyo et al. [33] developed chemically induced chromosomal evolution (CIChE) system which can integrate target genes into the chromosome with high copy numbers. However, chloramphenicol resistance marker was still employed. In 2013, a modified CIChE method that used triclosan as a selective marker was developed (Fig. 2a) [34]. By this strategy, a gene cluster containing deregulated *aroG*, *tktA*, *aroB* and *aroE* was integrated into the genome of *E. coli* BW25113. Combined with deletion of *aroK* and *aroL*, and integration of an additional chromosomal copy of *tktA*, *pntAB* and *nadK* under the control of 5 tandem repeats of the core-tac-promoter, the shikimate production of final strain SA116 increased obviously [23].

### Genetic circuits

By traditional metabolic engineering strategies, such as gene knockouts, expression level tuning, and protein engineering, industrial scale of desirable products can be produced by microorganisms. However, most of these non-native controllers are static which cannot sense changes of pathway output or cellular environment. Accordingly, any deviations away from the design conditions may result in decreased productivity [35]. To solve this problem, many genetic circuits have been desired to fine-tune and rebalance metabolic pathway, such as metabolic toggle switch [36, 37], biosensors [38, 39], and riboswitch [40, 41]. Although these genetic circuits are promising, there were only a few examples of their application in the field of bio-production, especially for shikimate production.

In most shikimate producers, shikimate kinase I and II respectively encoded by *aroK* and *aroL* were directly deleted to block downstream pathways, resulting in these engineered recombinant strains auxotrophic. To maintain normal growth, supplement of three aromatic amino acids and other aromatic compounds, such as p-hydroxybenzoic acid, potassium p-aminobenzoate and 2,3-dihydroxybenzoic acid, became necessary and thus increased the cost of industrial production [22]. To overcome this obstacle, we constructed a non-auxotrophic shikimate-producing strain of *E. coli* by a tunable switch, and this tunable switch can achieved conditional decreased expression of *aroK* (Fig. 2b) [12]. During the growth phase, *aroK* was maintained at a high expression level to achieve maximum biomass; and then, most of AroK activity was repressed by addition of an optimal concentration of inducer. In 5-L fed-batch fermentation, the final strain P-9 could produce 13.15 g/L shikimate without the addition of any aromatic compounds. This tunable switch also provided an effective tool for regulating indispensable genes involved in critical metabolic pathways. In 2017, Gupta et al. constructed a pathway-independent quorum-sensing circuit to dynamically regulate the expression of *aroK*, and the titers of shikimate in *E. coli* increased from unmeasurable to over 100 mg/L in minimal medium [42] (Fig. 2c).

Apart from tunable switch, growth dependent promoter can also be employed to construct a shikimate producing strain without completely blocking the aromatic amino acid biosynthesis pathway. In 2017, Lee et al. used promoters prpsM or prrnBP1 to regulate the *aroK* expression in shikimate producing *E. coli* [8]. Combined with overexpression of dehydroquinate dehydratase-shikimate dehydrogenase from woody plants, 5.33 g/L shikimate was accumulated in the resulting strain SK5/pSK6, demonstrating the effect of growth phase-dependent control of the *aroK* gene.

### Fluxomics and metabolomics

To increase flux of target pathway, manipulation of single or multiple genes are often used in metabolic engineering. However, unnecessary metabolic burden would be generated from over-engineering of the pathway, insufficient supply of precursors, or excessive deletion of competing pathways [2]. Fluxomics can achieve measurement of all metabolic reaction rates in a biological system. In other hand, metabolomics is a powerful tool for exploring the intricate biochemistry of cells in response to different conditions, such as stress or nutrition. By fluxomics and metabolomics, a global remodeling of the carbon and energy metabolism in shikimate producing strains can be exhibited. In 2017, by fluxomics and metabolomics analysis, Rodriguez et al. [43] analyzed an engineered shikimate-producing *E. coli* strain AR36 with a high-copy plasmid expressing six enzymes. It showed more glucose was consumed and directed into shikimate pathway in AR36 which avoided intermediates be converted into more toxic products. In addition, pentose-phosphate pathway in AR36 was strongly activated to supply E4P and balance the NADPH requirements for shikimate synthesis. From the results of fluxomics and metabolomics, novel candidate targets of genetic modification can be revealed to further improve the shikimate titer.

## Conclusions and perspectives

Microbial production of shikimate has been carried out for decades, and most producing strains were obtained by metabolic engineering of wild-type microorganisms, such as *E. coli*. Apart from glucose, other carbon source, such as glycerol [8, 44] and sorbitol [45], were also employed for shikimate production in *E. coli*. Up to now, the best *E. coli* strain can produce 84 g/L shikimate with a yield of 0.33 mol/mol glucose [20]. With the help of novel technologies, shikimate producing strain can be further optimized by dynamically regulated expression of *aroK*, and stably expression of rate-limiting genes on the chromosome. However, until now, only a few synthetic biology tools were successfully applied in shikimate production of *E. coli*. In *Corynebacterium glutamicum*, the clustered regularly interspaced short palindromic repeats interference (CRISPRi) has been applied to regulate gene expression at the transcriptional level and adjust the metabolic flux in shikimate synthetic pathway. The titers of shikimate reached 7.76 g/L in 250 mL flasks and 23.8 g/L in 5-L fermentor [46]. In addition, by an aerobic, growth-arrested and high-density cell reaction, the shikimate production in recombinant *C. glutamicum* could achieved a titer of 141 g/L and a yield of 51% (mol/mol) from glucose [47]. In the future, CRISPRi and growth-arrested cell reaction can also be attempted in shikimate production of *E. coli*.

It is noteworthy that most shikimate producers were obtained by rational metabolic engineering, which relies on the genetic tools available for target microorganism. However, for non-model microorganisms, the development of novel genetic tools is costly and time-consuming. As a result, shikimate producers were almost restricted to *E. coli*, and only a few attempts were conducted in *Bacillus subtilis* and *C. glutamicum*. In another hand, the classical approach based on random mutagenesis is often a viable method to improve the phenotype of non-model strains. Development of an effective screen method of shikimate may facilitate the application of this classical approach in engineering other shikimate producers. By transcriptome analysis, a biosensor sensitive to shikimate may be obtained, which can facilitate the engineering of a shikimate producer. This method has been successfully applied to the discovery of 1-butanol sensors [48]. In addition, l-tyrosine production in *E. coli* was linked with synthesis of the black and diffusible pigment melanin by introducing a tyrosinase, and an effective screen method was developed in isolating l-tyrosine overproducing strains [49]. Similarly, if shikimate can be transformed into a chemical compound with color or other detected signals, screen of shikimate producers will be conveniently and quickly.

## Abbreviations

NADPH: nicotinamide adenine dinucleotide phosphate
G6P: glucose-6-phosphate
F6P: fructose-6-phosphate
F1-6P: fructose-1, 6-bisphosphate
G3P: glyceraldehyde-3-phosphate
6PGL: 6-phosphoglucono-lactone
6PG: 6-phosphogluconate
2D3D6PG: 2-dehydro-3-deoxy-d-gluconate-6-phosphate
RU5P: ribulose-5-phosphate
X5P: xylulose-5-phosphate
R5P: ribose-5-phosphate
S7P: sedoheptulose-7-phosphate
E4P: erythrose-4-phosphate
PEP: phosphoenolpyruvate
PYR: pyruvate
ACoA: acetyl coenzyme A
OAA: oxaloacetic acid
DAHP: 3-deoxy-d-arabino-heptulosonate-7-phosphate
DHQ: 3-dehydroquinate
DHS: 3-dehydroshikimate
S3P: shikimate-3-phosphate
CHA: chorismate
EPSP: 5-*enol*pyruvylshikimate-3-phosphate
l-Phe: l-phenylalanine
l-Tyr: l-tyrosine
l-Trp: l-tryptophan
CIChE: chemically induced chromosomal evolution
AHL: 3-oxohexanoylhomoserine lactone
